# Practice Patterns and Perspectives on Stereotactic Body Radiation Therapy for the Metastatic Spine From Lower- and Middle-Income Countries

**DOI:** 10.1200/GO.22.00167

**Published:** 2022-09-14

**Authors:** Rima Sanjay Pathak, Anil Tibdewal, Rajesh Kinhikar, Katherine Wakeham, Kamal Akbarov, Lisbeth Cordero, Ashwini Khandavalli, Jai Prakash Agarwal

**Affiliations:** 1 Department of Radiation Oncology, Tata Memorial Centre, Mumbai, India; 2 Medical Physics, Tata Memorial Centre, Mumbai, India; 3 Applied Radiation Biology and Radiotherapy Section, Division of Human Health, Department of Nuclear Sciences and Applications, International Atomic Energy Agency, Vienna International Centre, Vienna, Austria; 4 Applied Radiation Biology and Radiotherapy Section, Division of Human Health, Department of Nuclear Sciences and Applications, International Atomic Energy Agency, Vienna, Austria

## Abstract

We wanted to understand the current practice patterns and stereotactic body radiation therapy (SBRT) utilization for spine metastases in lower- and middle-income countries (LMICs).

**METHODS:**

A questionnaire was designed to identify the current practice patterns of treating spine metastases, uptake of spine SBRT in routine care, dose fractionations commonly used, and the perceived benefits and toxicities of using ablative doses. Individuals registering for a spine SBRT workshop were requested to answer the questionnaire.

**RESULTS:**

We received 395 responses from radiation oncologists (ROs) working in 12 different LMICs. The majority of respondents were from an academic institute (57.5%). Two hundred seventy-four respondents further identified themselves from the government/public sector (44.89%), corporate/private sector (47.89%), not-for-profit organization (5.4%), or public-private partnership (5.4%). The respondents indicated that 8.43%, 27.46%, 41.73%, and 10.04% of the spine metastases patients are treated using clinical marking, X-ray–based, 3D conformal radiation therapy, and SBRT, respectively. A third of the respondents did not have any experience of spine SBRT; those with high-volume practice were predominantly from an academic institute. The majority of respondents would use spine SBRT to reduce pain severity (71.9%) and achieve durable pain control (61.01%) in the setting of oligometastases (92.73%) and reirradiation (56.69%). Respondents preferred 3- to 5-fraction regimens (64.9%) over 1-2 fractions (33.68%). The top three reasons for not using spine SBRT were resource constraints (50%), lack of machine (37.11%), and lack of training (27.34%).

**CONCLUSION:**

There is heterogeneity in spine SBRT practice and utilization between academic and nonacademic institutes. Resource and infrastructure constraints along with lack of training are limiting the use of SBRT among ROs from LMICs. Collaborative studies from LMICs will help in resolving unique challenges posed by resource constraints.

## INTRODUCTION

Palliative radiation therapy (RT) has remained the mainstay of treatment for spine metastases and is traditionally delivered in a single fraction or with a protracted regimen producing a durable response in 60%-70% of patients, whereas the others might have recurrent symptoms requiring reirradiation.^1^ With new advances in imaging modalities, small volume metastases to limited sites called oligometastases are detected more frequently than in the past. Such individuals have longer survival with ablative oligometastasis-directed therapy than patients with widespread disease.^2^ Complementing the imaging refinement, the improved radiation planning allows precise delivery of very high doses of radiation in a single or small number of fractions to an extracranial target within the body, called stereotactic body radiation therapy (SBRT).^3^ The quantum of evidence favoring the use of SBRT is growing rapidly.^4^

**CONTEXT**

**Key Objective**
We wanted to understand the current practice patterns and stereotactic body radiation therapy (SBRT) utilization for spine metastases in lower- and middle-income countries (LMICs) through a questionnaire.
**Knowledge Generated**
Three hundred ninety-five radiation/clinical oncologists from 12 LMICs through their responses indicated that only approximately 10% of patients with spine metastases undergo SBRT. SBRT was mostly used in the setting of oligometastatic disease and reirradiation essentially to achieve better and durable pain control along with its progression-free survival benefit. Significant heterogeneity in access to the technology, resource constraints, and knowledge was among the most common reasons for underutilization of SBRT spine across all LMICs.
**Relevance**
Our study helped to identify practice patterns for treatment of spine metastases and the issues with SBRT spine implementation in LMICs. Cooperation within the international organizations with a focus on the implementation of SBRT on the basis of the results of such collaborative studies may help to overcome barriers.

A nationwide survey using the US National Cancer Database (NCDB) showed that by 2013, the utilization of spinal SBRT increased from 2% to 20%.^5^ Another publication from NCDB reported that the SBRT utilization varied by patient factors including race, insurance, institute location, and whether the cancer center was an academic institute.^6^ This variation in utilization may be due to the resource-intensive nature of SBRT, and more pronounced disparity in the uptake could be present among lower- and middle-income countries (LMICs). Recently, two randomized studies, RTOG 0631 and CCTG/TROG SC.24, have reported conflicting results where the former showed no improvement, whereas the latter showed significant improvement in pain control with SBRT compared with conventional RT for painful bone metastases.^7,8^ It is unclear how these results have changed the perceptions and practice among radiation oncologists (ROs).^7,8^

Delivering ablative doses of radiation can lead to severe toxicities and even treatment-related death.^2^ Thus, implementation without appropriate training is discouraged. There are very few holistic training programs for stereotaxy for ROs, medical physicists, dosimetrists, and radiation therapists especially in LMICs, which may limit its utilization. There is a paucity of published reports from LMICs on the utilization and challenges encountered during patient selection, treatment planning, and delivery of spine SBRT. Thus, to understand the current practice patterns in LMICs on treating patients with spine metastases with SBRT, we conducted a study using a questionnaire.

## METHODS

The questionnaire contained 18 questions designed to identify the current practice of treating patients with spine metastases, uptake of SBRT in routine care, modalities of imaging and frequency, commonly used dose fractionation, perceived benefits, and toxicities seen in patients. A virtual spine SBRT workshop was organized by the radiation oncology department of the Tata Memorial Centre, Mumbai, in September 2021. Interested individuals were required to answer the questionnaire for registering in the workshop. The link for answering the questionnaire was active for 28 days, sent to the oncologists' e-mail, and also advertised on the website of the Association of Medical Physicists of India (AMPI) and social media platforms. The responses were collated using custom-built software. Duplicate entries were identified using the unique e-mail IDs that participants registered with.

The respondents were grouped by the type of institute they practiced in. Most LMICs have a similar health care structure wherein the government hospital is synonymous with public sector hospital where treatments and investigations are subsidized by the government to help underprivileged patients and are not driven to generate profits. Contrarily, the corporate hospitals are synonymous with the private sector hospitals, which are profit-oriented and essentially serve the privileged patients or those with insurance. Since a large majority of respondents were from India, we performed comparisons among the responses from India with those from other countries. Furthermore, we grouped the variables on the basis of their median or mean values wherever appropriate for comparisons, which have been reported using descriptive statistics. Intergroup comparisons were made using the chi-square test for categorical variables and an independent t-test for comparing means, and a *P* value ≤ .05 was considered significant. The analysis was performed using the IBM-SPSS software v.22.

## RESULTS

We received a total of 440 responses, of which 395 were from 12 countries classified as LMICs by the world bank (Appendix Table A1). Twelve responses from non-LMIC ROs, and 33 duplicate responses were eliminated during analysis. We compared the responses provided by the participants from countries other than India with those received from Indian participants to evaluate if we could analyze the data together. We found that they were similar across all domains except that none of the participants from other countries had a high-volume SBRT spine practice (> 30 cases/year) and the proportion of respondents who reported resource constraints as their barrier to spine SBRT utilization (Appendix Table A2). Thus, moving forward, the results report responses from the all the countries together.

The majority of respondents (n = 227, 57.5%) worked in an academic institute. The question asked the respondents to select all that apply (choices provided were government/public, corporate/private, public-private partnership, not-for-profit, Academic, and nonacademic) for their institutional affiliation, but nearly a third of them (n = 121, 30.6%) did not classify beyond the academic institute. Among the remaining 274 respondents, 123 (44.89%) worked in a government institute, whereas 131 (47.8%) worked in a corporate/private hospital setup. Few respondents worked in institutes that were not-for-profit (n = 15, 5.4%) or with a public-private partnership (n = 5, 1.8%). The practice patterns of the respondents are detailed below.

### Techniques Used for Treating Spine Metastases

In terms of radiotherapy practice in LMICs, an average of 8.43% of spine metastases patients are treated with clinical marking and 27.46% with 2-dimensional (2-D)/X-ray–based planning. The majority of respondents (41.73%) preferred using 3D conformal radiation therapy (3DCRT). More resource-intensive techniques such as intensity-modulated radiation therapy (IMRT) and SBRT are used relatively infrequently on average in 12.15% and 10.04% of patients, respectively. Table 1 summarizes the proportion of patients treated using different techniques by the type of institute and the comparisons among them. Comparing academic with nonacademic and government with corporate institutes, we found that academic and government institutes treated a significantly higher proportion of patients with clinical or 2-D planning and a significantly lower proportion with the IMRT technique (Table 1). Contrarily, a significantly higher proportion of patients at corporate institutes were treated with a resource-intensive technique like SBRT.

**TABLE 1:**
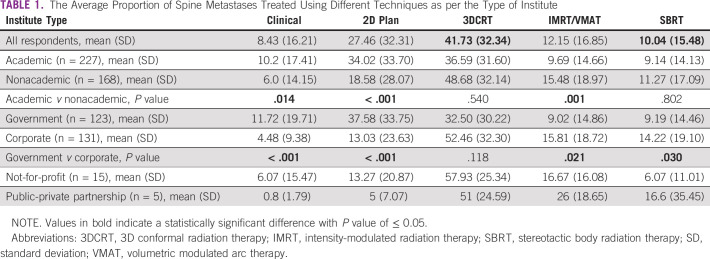
The Average Proportion of Spine Metastases Treated Using Different Techniques as per the Type of Institute

Since an individual's proficiency and experience can affect practice patterns, we asked the respondents to indicate the approximate number of spine metastases treated with SBRT during the past 1 year (Table 2). Nearly a third of the respondents had not treated any patient with SBRT, and a little over a third had treated five or fewer. Respondents with a low-volume SBRT practice (0 to ≤ 5) were similarly distributed between academic and nonacademic institutes. However, higher-volume practice (> 15 to ≤ 30 SBRTs annually) was significantly more likely to be from academic institutes (*P* = .001). Similar comparisons between government and corporate institutes showed that a significantly higher percentage of respondents from government institutes (*P* < .001) had no experience in SBRT, whereas a higher proportion of corporate respondents had a moderately high experience of > 15 to ≤ 30 SBRTs annually (*P* = .012).

**TABLE 2:**
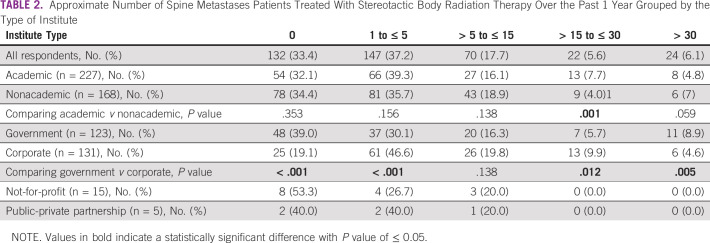
Approximate Number of Spine Metastases Patients Treated With Stereotactic Body Radiation Therapy Over the Past 1 Year Grouped by the Type of Institute

### Indications and Perceived Benefits of Spine SBRT

**FIG 1:**
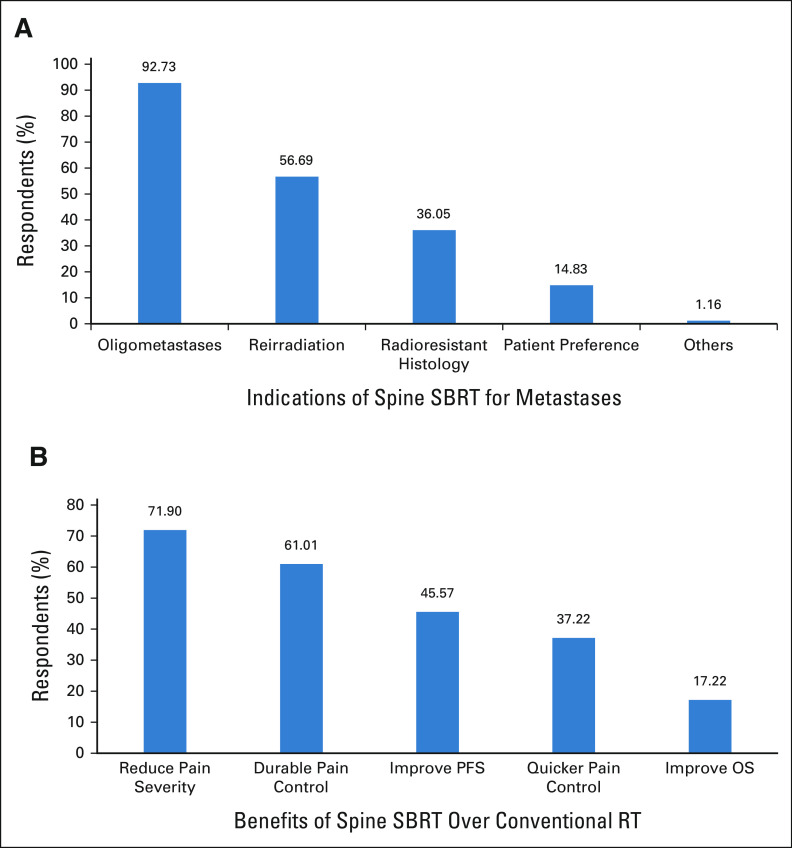
(A) Indications and (B) perceived benefits of SBRT for spine metastases selected by the respondents. OS, overall survival; PFS, progression-free survival; SBRT, stereotactic body radiation therapy.

Among the 395 respondents, 48 (12.2%) did not choose any of the provided options for indications of spine SBRT. Figure 1A shows the preferred indications of spine SBRT that were chosen by the remaining. The top three indications selected were oligometastatic disease (92.73%), reirradiation of the spine segment (56.69%), and metastases from radioresistant histology (36.05%). Significantly higher respondents from corporate institutes used SBRT for radioresistant histology (35.9% *v* 25.2%, *P* < .001) and patient preference (13% *v* 5.7%, *P* < .001). The top three perceived benefits selected were reduction in severity of pain (n = 284, 71.9%), greater durability of pain control (n = 241, 61.01%), and improvement in progression-free survival (PFS; n = 180, 45.57%) as seen in Figure 1B. Significantly fewer respondents from the academic institutes selected improvement in overall survival (15.4% *v* 19.6% *P* = .030) and quicker pain response (34.8% *v* 40.5%, *P* = .029), whereas significantly higher respondents selected improvement in PFS (49.8 *v* 39.9%, *P* = .002).

### The Practice of Spine SBRT

Delineation of the spinal cord for spine SBRT was carried out by coregistering magnetic resonance imaging (MRI; n = 179, 53.4%), computed tomography (CT) myelogram (n = 12, 3.58%), MRI with CT myelogram (n = 21, 6.27%), noncontrast CT scan (n = 31, 9.25%), MRI with noncontrast CT scan (n = 78, 23.28%), or the combined use of all three imaging modalities (n = 10, 2.99%). Similarly, we also asked the respondents to indicate their preference for the clinical target volume (CTV) contouring guideline. The majority of them use the international spine radiosurgery consortium guidelines^9^ (n = 209, 69.66%), followed by no CTV (n = 39, 13%), institutional guideline (n = 38, 12.66%), or contouring the entire vertebra irrespective of the gross disease (n = 14, 4.66%). There was no difference in the contouring preferences among oncologists from different types of institutes.

Significant heterogeneity exists in the preferred spine SBRT dose fractionation across the globe. Therefore, we asked the respondents their most commonly used dose and fractionation regimen. Of the 285 (72.15%) who answered, the majority preferred 24-27 Gy/3 fr (n = 98, 34.38%), closely followed by 30-35 Gy/5 fr (n = 87, 30.52%). Single-fraction (18-24 Gy) or 2-fr regimen (20-24 Gy) was preferred by relatively fewer respondents (n = 47 and n = 49, respectively). The ROs from academic institutes showed a clear preference for the 2-fr regimen (15% *v* 8.9%, *P* < .001), whereas those from nonacademic institutes preferred the 5-fr regimen (18.1% *v* 27.4%, *P* = .028). In similar comparisons between those from government versus corporate institutes, we found that a significantly higher percentage of respondents from government institutes preferred the ≤ 2-fr regimen (44% *v* 25.9%, *P* < .001).

### Toxicities of Spine SBRT

The questionnaire specifically asked the respondents to select the post-SBRT toxicities experienced by their patients. Only 270 (68.35%) respondents answered this question. The top three toxicities reported pain flare (n = 135, 50%), no toxicity (n = 74, 27.41%), and vertebral compression fractures (n = 60, 22.22%). We wanted to see if there was any association between the number of SBRT performed per year and toxicity experienced. We found that a significantly higher percentage of respondents, who perform > 15 spine SBRTs per year, had seen their patients experience VCF (23.9% *v* 16.1%, *P* = .020), whereas significantly higher respondents with low-volume practice (< 15 spine SBRT per year) reported no toxicity (27.2% *v* 19.6%, *P* = .019). We also compared the preferred fractionation regimen (1-2 fr *v* 3-5 fr) with the toxicities and found that VCF (29.5% *v* 14.5%, *P* < .001) was associated with respondents using ≤ 2 fr, whereas radiation neuropathy (9.5% *v* 14.5%, *P* = .014) was reported more frequently by respondents using > 2 fr.

### Barriers to SBRT Spine Utilization

**FIG 2:**
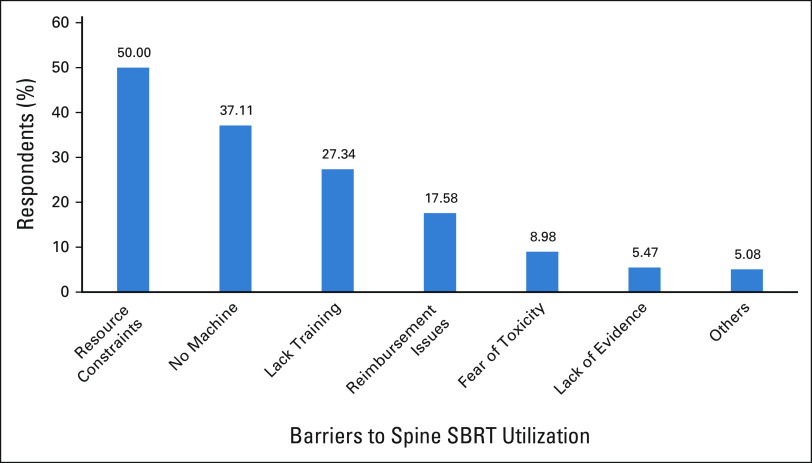
Reasons indicated by the respondent for not using SBRT for spine metastases. SBRT, stereotactic body radiation therapy.

Finally, to understand the reservation of the respondents who had not treated patients or rarely treat patients with spine metastases using SBRT, we requested these respondents to select the reason(s) from a list provided and free text if needed. Nearly a third of the total respondents mentioned that they had no reservations (n = 139, 35.2%). The top three reasons for not using SBRT (Fig 2) were resource constraint (50%), appropriate machine not available (37.11), and lack of training in SBRT for spine metastases (27.34%). Only 5.08% indicated that they felt that the evidence in support of using SBRT for spine metastases was inadequate. Resource constraints were reported significantly more often by respondents from other countries as compared with those from India (Appendix Table A2). This was among the very few statistically significant differences noted between the responses from India and those from other countries.

## DISCUSSION

Our publication reporting on the current trends in acceptance and utilization of spine SBRT in LMICs addresses the paucity of knowledge in this field. We showed that in LMICs, nearly 35% of spine metastases patients are planned using nonconformal methods, including clinical or 2-D/X-ray–based marking. This was found to be heavily influenced by the respondents from the academic and the government institutes possibly because of resource availability, constraints, and resource allocation for palliative treatments on the basis of the perception of cost-effectiveness.^10^ Our report also lays bare the inequity in resource utilization/allocation between the public and the private sector, which is a surrogate for the different socioeconomic groups in LMICs.^11^ SBRT for spine metastases is being used for about 10% of the cases.

The ASTRO guideline on palliative RT for bone metastases predated the recent reports from the randomized studies RTOG 0631 and CCTG SC.24, which suggest that SBRT should be offered in a clinical trial setting with great emphasis on adherence to the quality and safety criteria laid in the ASTRO white paper.^7,8,12^ We did not ascertain in this survey if SBRT was being recommended only under the trial setting or as a part of standard practice. The RTOG 0631 study showed that compared with conventionally fractionated RT, using 16-18 Gy/1 fr spine SBRT showed no difference in the change in pain response (mean change in pain score –3.83 *v* –3.0) or overall pain response (57.9% *v* 40.3%, *P* = .99).^7^ On the contrary, a similar study CCTG SC.24 showed that higher biologically equivalent doses of SBRT to the spine (24 Gy/2 fr) significantly improved the complete response rates for painful metastases at 3 months (14% *v* 35%, *P* < .001) and this difference was maintained at 6 months (16% *v* 32%, *P* = .004).^8^ These results might have been a contributing factor to the interest in our workshop and participation in this survey.

We also report the relative lack of experience in treating spine with SBRT as a third of respondents had not treated any patient with spine SBRT in the past 1 year. This could also stem from sampling bias as the questionnaire was directed at those who were interested in attending a workshop on spine SBRT. The remaining two thirds of the respondents had treated at least ≥ 5 patients with spine metastases with SBRT. This showcases the growing interest in radiosurgery for extracranial sites and has been previously reported from the United States and a similar study from India.^13,14^ There is a lack of randomized evidence of reirradiation setting or that for radioresistant primary, but retrospective studies indicate that feasibility, safety, and efficacy of this approach may be contributing factors for respondents preferring to use SBRT for these clinical scenarios.^15-18^ Several benefits of SBRT for spine metastases have been demonstrated via prospective and randomized studies such as improved PFS, overall survival, and durability of pain control, and these were appropriately selected by the respondents.^2,8,15,19,20^ With the increasing evidence in favor of SBRT spine for painful and oligometastatic disease, the utilization will increase and greater emphasis should be laid on uniform treatment policies, safety, and quality assurance.

An early formulation of the ISRS consensus guideline in 2012 might have been the reason for the majority of respondents (69.66%) using it for contouring the CTV and preferring to use MRI (85.94%) for delineating the cord.^9^ However, MRI may not be readily available or affordable for all the patients receiving SBRT in LMICs where a CT myelogram can be used instead. It is increasingly recognized that single fraction treatments, especially in lytic spinal metastases, have a higher probability to produce a VCF.^21-23^ Thus, more centers, especially academic centers, are choosing the 2-fr regimen to balance the efficacy of hypofractionating and the comfort of delivering the treatment in the least number of fractions. Respondents from busy government institutes also leaned toward using 1-2 fractions probably to economize on the machine time. Different fractionation can be associated with different side effects, and this was also seen in our study. In addition, toxicities are a probabilistic event, and therefore, the respondents with higher-volume practice had seen patients experience several different toxicities as compared with those who performed < 15 spine SBRTs per year. Nevertheless, the responses were based on the individual respondents recall and therefore have to be interpreted with caution.

Our study showed the substantial gap between the radiotherapy requirements and the present availability of SBRT in LMICs. The lack of resources, machines, and training was among the top deterrents to the implementation of SBRT. Issues that are commonly faced in India were similarly echoed by those from other LMICs. The necessity of appropriate actions to meet the needs of LMICs in the strengthening of SBRT application is obvious. One of them is networking and support of the radiotherapy centers at the international level. An example could be the International Atomic Energy Agency initiative to support LMICs under Regional Cooperative Agreement for Research, Development and Training Related to Nuclear Science and Technology for Asia and the Pacific (RCA) region. Two projects focusing on the implementation of SBRT in 21 countries of the RCA region have already been run. The gap in knowledge can further be reduced through student exchange or international fellowship programs that can upon returning provide training to others and encourage safe and appropriate infrastructure and equipment utilization. Bringing automation in various processes of simulation, planning, and treatment of SBRT can help to reduce interpersonal variability and reduce the cost of treatment and improving access among LMIC patients.

Although simplistic in the design, there are several limitations to this study that are associated with a questionnaire-based approach that relies on the respondent to provide accurate information. This can be fraught with several biases such as recall, sampling, nonresponse, and conformity bias, among others. Nevertheless, during the creation of the questionnaire, we tried to minimize biases like the order bias by randomly appearing multiple-choice responses and choosing the question order in a manner where an individual is appropriately primed to answer the subsequent question. We mitigated the acquiescence bias by phrasing the questions in a neutral manner, which also helped to reduce the conformity bias. Since the questionnaire did not require the respondents to provide identifying details like their name or the hospital they work in, we ensured that nonresponse bias because of reluctance was mitigated. Nevertheless, biases like the inherent selection bias of individuals who responded were interested in learning SBRT and were chiefly from India have to be kept in perspective when interpreting the results. The results can significantly be influenced by the years in practice and formal training in SBRT, but this information was not available. The strengths of the study lie in the high number of responses from radiation/clinical oncologists practicing in the LMICs with varying levels of expertise and from different types of institutes.

In conclusion, the questionnaire was successful in highlighting the barriers to the utilization of spine SBRT. There is growing evidence in support of spine SBRT for at least oligometastatic and painful spine disease, indicating that the demand for such procedures will rise in the future. The current heterogeneity in practice and access across the different types of institutes needs to be addressed by the policymakers. Similar responses from the participants from India and other LMICs indicate common socioeconomic and infrastructural issues with the existing health care structure. Collaborative studies should be conducted to pool data from various institutes across the LMICs to help resolve the unique challenges posed by resource constraints. Cooperation within the international organizations with a focus on the implementation of SBRT on the basis of the results of such collaborative studies is one of the effective ways to overcome barriers.

## Figures and Tables

**TABLE A1:**
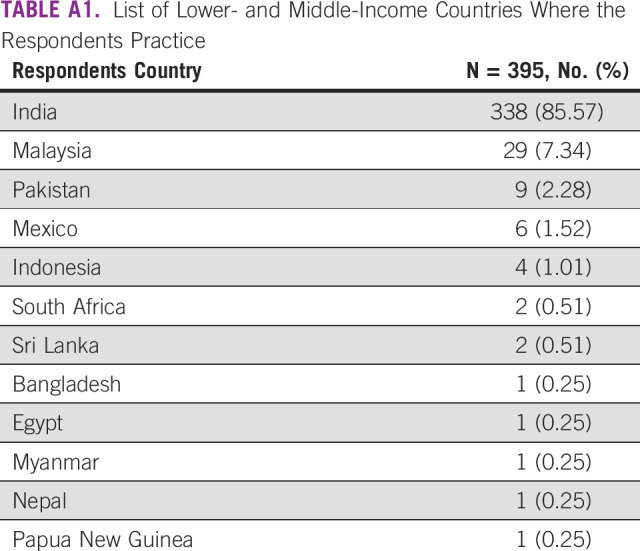
List of Lower- and Middle-Income Countries Where the Respondents Practice

**TABLE A2:**
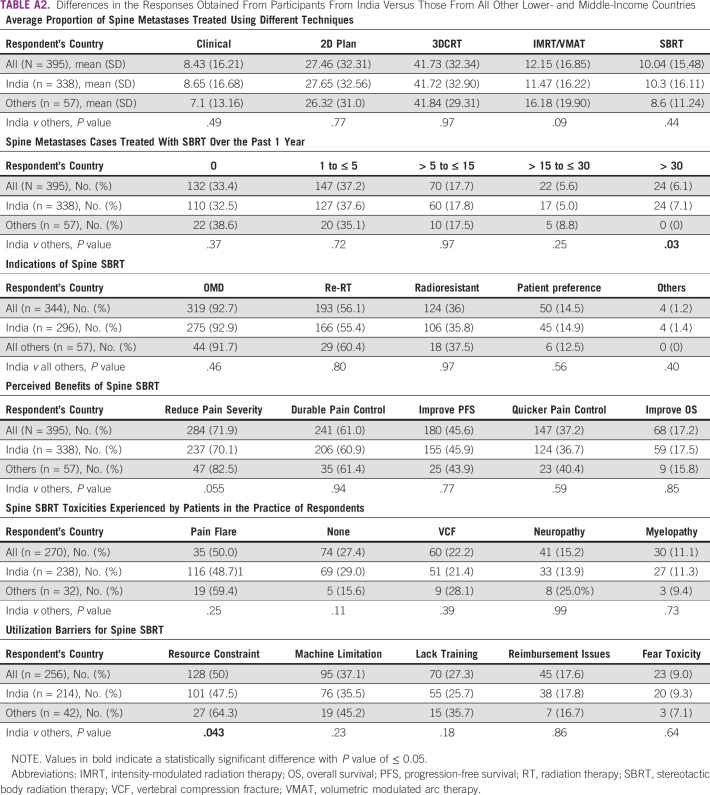
Differences in the Responses Obtained From Participants From India Versus Those From All Other Lower- and Middle-Income Countries
